# Factors related to cognitive function in type-2 diabetes and neuropathic pain patients, the role of mood and sleep disorders in this relationship

**DOI:** 10.1038/s41598-022-18949-4

**Published:** 2022-09-14

**Authors:** Jenifer Palomo-Osuna, María Dueñas, Cristina Naranjo, Helena De Sola, Alejandro Salazar, Inmaculada Failde

**Affiliations:** 1 Observatory of Pain, Grünenthal Foundation-University of Cadiz, 11009 Cádiz, Spain; 2 Biomedical Research and Innovation Institute of Cadiz (INiBICA), 11009 Cádiz, Spain; 3 Preventive Medicine and Public Health Area, University of Cádiz, 11009 Cádiz, Spain; 4 Department of Statistics and Operational Research, University of Cádiz, 11510 Puerto Real, Spain; 5 University Hospital Puerta del Mar, 1009 Cádiz, Spain

**Keywords:** Diabetes, Psychology

## Abstract

To compare cognitive function in patients with diabetes mellitus type-2 (T2DM) both with and without diabetic neuropathic pain (DNP). To analyse the relationship between mood and sleep disorders, quality of life and cognitive function in patients with DNP. Cross-sectional study conducted in patients with T2DM and neuropathy. The presence of DNP, cognitive function, mood status, sleep quality, health-related quality of life, pain intensity and phenotype of pain were measured. Descriptive, bivariate and multivariate analyses were performed. A total of 149 patients (71 with DNP) were included. Patients with and without DNP presented similar scores on the TYM (41.46; SD = 6.70 vs. 41.97; SD = 5.50) and those with DNP had a slightly higher frequency of cognitive impairment (TYM score ≤ 41: 40.8% vs. 43.6%). The patients without DNP performed better in the verbal fluency dimension (mean = 3.53; SD = 0.98 vs. mean = 3.82; SD = 0.66). Being older (B = − 0.258) and under treatment with insulin (B = − 2.919) were related with greater cognitive impairment. Obesity (OR = 17.277) and a longer duration of diabetes (OR = 1.317) were also related to greater risk of cognitive impairment. Impaired cognitive function in patients with DNP is more related to T2DM factors than pain factors. The presence of depression and a worse quality of life were related to a greater risk of cognitive impairment. Identifying and controlling these factors should be an essential intervention for maintaining the cognitive function in patients with T2DM and DNP.

## Introduction

Cognitive impairment is an important public health problem that affects between 6.7 and 25.2% of the worldwide population over 60 years of age^1^ and between 12.4 and 16.8% of Spanish adults over 65 years old^2^. The presence of this impairment has been related with a wide range of symptoms, such as loss of memory, mood and behaviour changes, and motor and speech disorders^1,2^. These are all causes of disability and have a great impact on the quality of life^3^. Besides older age, a number of factors have been associated with a higher risk of cognitive impairment in different groups of patients, the main ones being a low socio-economic level, a limited social life, genetic factors, endocrine disorders and brain injuries^1,2^.

The risk of cognitive impairment has been observed to be 1.5 times higher in patients with diabetes mellitus type 2 (T2DM) than in individuals without this pathology^4^. However, T2DM often presents with other comorbidities that are also related with cognitive impairments, making it difficult to establish a causal relationship between these processes^5^.

Chronic pain (CP) is another condition related with cognitive impairment^6,7^. Patients with CP have been reported to often suffer from losses of concentration, memory problems and impaired processing speed and attention^7^. Moreover, CP patients have been shown to present affected subcortical areas, similar to those detected in patients with mild cognitive impairment^8^. One of the most common causes of CP is peripheral neuropathic pain. Patients with this pathology have been reported to suffer from changes in the neural pathways involved in cognition as a result of the pain^9^. Likewise, the damage caused in the peripheral nerve in painful neuropathy leads to maladaptive responses in the somatosensory system, which produces different responses, including sensory losses, hypersensitivity, spontaneous pain (ongoing), allodynia and hyperalgesia (evoked pain)^10^, which could affect the cognitive functioning of the patients in a variety of ways.

Diabetic neuropathy is a common complication associated with T2DM^9^, affecting around 50% of the patients. Although its causes are unknown, the most supported theory is that the damage nerve cells is caused by oxidative and inflammatory stress in a context of metabolic dysfunction^11,12^. In addition, approximately 15–25% of these subjects suffer from Diabetic Neuropathic Pain (DNP)^9^. Considering this scenario, and that both CP and diabetes are associated with cognitive impairment, it seems reasonable to think that cognitive impairment could be particularly frequent in patients suffering from diabetic neuropathic pain. However, the study of cognitive function in patients with DNP has received little attention^13,14^. Supporting this potential relationship, some studies have shown that patients with DNP have a lower grey matter volume than diabetic patients without DNP^15^.

On the other hand, a recent systematic review has shown that anxiety and depression are present in almost a quarter of patients with DNP and nearly 50% suffer from sleep disorders^16^. Also, several studies have shown that these disorders are most common in patients with DNP than in patients without DNP, and it has been related to presence of pain that cause a great disability^14,17^. It has been shown that depression and anxiety are associated with chronic diseases, such as diabetes or chronic pain, due to common biological mechanisms including chronic inflammation, neuroendocrine dysregulation, oxidative stress or mitochondrial dysfunction, among others. All of them are related to psychosocial stress^18^. Likewise, it is well known that sleep disorders have a strong relationship with CP^19^. Haak et al., have shown that sleep deficiency has a de-activating effect on several systems with predominantly analgesic properties, like opioids, and melatonin^19,20^. Therefore, analysing the relationships between mood and sleep disorders and cognitive function in patients with DNP could be of great interest.

Taking all of the above into consideration, this study aims to compare cognitive function in patients with T2DM with and without DNP, and analyse the relationship between mood disorders, sleep disorders and quality of life and cognitive function in patients with DNP. Due to the close relationship between pain and cognition, we hypothesized that patients with T2DM and DNP will have worse cognitive function that patients without DNP. Likewise, we hypothesized that the presence of mood and sleep disorders and a worse quality of life will have a negative impact on the cognitive function of patients with DNP.

## Methods

### Subjects

A multi-centred cross-sectional study was conducted between June 2017 and March 2020 in six primary care centres in Cádiz (Andalusia, Spain), including patients over 18 years of age diagnosed with T2DM according to the criteria of the American Diabetes Association (ADA)^21^, and who were included in the “*Diabetes Mellitus Integral Care Process”* (PAIDM). The presence of diabetic neuropathy in these patients was confirmed by a clinical foot examination using the monofilament test^22^.

Patients were excluded from the study that did not meet the inclusion criteria, those presenting any kind of neurodegenerative disease with cognitive impairment, those that were unable to complete the scales due to a physical limitation, and when neuropathy was ruled out by the foot examination.

The study was approved the fifth of December 2020 by the Clinical Research Ethics Committee of the “Puerta del Mar” University Hospital (Cádiz, Spain) (Reference Number of the study: 1401-N-20), ensuring compliance with the standards of good clinical practice, and it was performed in accordance with the Helsinki Declaration.

### Population and sample

Taking as a reference data from the primary care centres where around 2000 patients with T2DM were included in the PAIDM of the DIRAYA system (an electronic system of medical records available in the Andalusian Health Service), and considering the expected prevalence of diabetic neuropathy to be approximately 25%, the number of people with T2DM and neuropathy was estimated to be 500. Likewise, from this population of 500 subjects, and considering the expected prevalence of DNP to be about 26%^9^, a sample size of 186 patients was calculated, according to the sample size calculation to estimate a proportion, with a 95% confidence level and precision of 5%.

Since the population with neuropathy had not been identified, patients were recruited using a consecutive non-random sampling technique based on the selection of high-risk patients, using four indicators of poor control of the disease for their selection: Hba1c > 8% in the last year; the presence of diabetic retinopathy; the existence of foot ulcers; having been diagnosed with diabetes over 10 years earlier. Patients were included in the study if they fulfilled at least one of these criteria.

The selected patients were contacted by telephone, the characteristics and aims of the study were explained to them and they were invited to take part. They were also informed of the need to sign the informed consent and of their right to abandon the study at any time. The subjects that agreed to participate were given an appointment at their corresponding primary care centre and were informed in detail of the procedure to be followed in the study.

Two researchers performed the clinical interview and the foot examination with the monofilament test according to the standard procedure. This procedure consists in the exploration of 10 reference points in each foot, 8 plantar and 2 dorsal. The diabetic neuropathy was confirmed when the patient did not perceive the monofilament in 3 or more points in one of their feet^23^. After confirming the presence of diabetic neuropathy, and according to the results of the “Douleur Neuropathique 4 (DN4)” scale, these patients were classified into two groups, with or without neuropathic pain. The DN4 scale has been widely used and consists of 10 binary items providing a final score from 0 to 10, with subjects scoring 4 or above being identified as presenting with DNP. This scale has been adapted and validated in Spanish and has a sensitivity of 79.8% and a specificity of 78.0%^24^.

### Instruments

Medical records and a structured questionnaire were used to gather sociodemographic (age, sex, educational level, and employment status) and clinical information such as time since diagnosis of T2DM, duration of DNP, treatment with insulin, a history of anxiety and depression and last level of glycated haemoglobin (HbA1c). Information was also collected about the presence of T2DM complications (retinopathy, nephropathy, diabetic foot and cardiovascular disease), the presence of cardiovascular risk factors (obesity, arterial hypertension (AHT) and dyslipidaemia) and whether they were taking analgesic or hypnotic medication or one affecting cognitive function; these were identified from a previously prepared list including drugs with the potential to affect cognition.

Cognitive function was assessed using the Test Your Memory (TYM) instrument, which evaluates 10 cognitive domains: orientation, copying, retrograde and anterograde memory, calculation, verbal fluency, similarities, naming objects, visuospatial ability and executive function. In this scale, by adding the scores from the 10 domains, a global score between 0 and 50 is calculated, a higher score corresponding to better cognitive performance. A cut-off point has been reported for this scale that enables subjects to be classified with cognitive impairment if their score is ≤ 41 points^25,26^^.^ This scale has been translated, adapted and validated in Spanish in patients with chronic pain by our research group^27,28^.

Pain intensity was measured using a visual analogue scale (VAS) with values ranging from 0 to 10, where 0 corresponds with no pain and 10 with the worst possible pain. The researchers asked the subjects to inform only about the presence of neuropathic pain, after passing the DN4 questionnaire where the symptoms were clearly described.

Anxiety and/or depression were measured using the Hospital Anxiety and Depression Scale (HADS), which consists of two sub-scales with seven items each: HADS-A (anxiety) and HADS-D (depression). Each item scores between 0 and 3 points and each sub-scale between 0 and 21, a score above 10 on either sub-scale indicating the presence of a state of anxiety and/or depression. This instrument has been validated in patients with diabetic neuropathic pain^29^ and in the Spanish population^30,31^.

The Medical Outcomes Sleep (MOS) scale was used to measure sleep quality. This instrument has been validated in Spanish and has appropriate psychometric properties for assessing sleep characteristics in patients with DNP. The instrument includes 12 items that examine the impact of the disease on the dimensions of sleep, and a summary index (Index-9) that measures sleep quality. Scores range from 0 to 100, higher scores indicating more sleep problems^32^.

Health-related quality of life was measured with version 2 of the SF-12 Health Survey (SF-12v2)^33^. This instrument includes 12 items that make it possible to calculate the profile of eight dimensions (physical functioning, role functioning, bodily pain, perception of general health, vitality, social functioning, emotional role functioning and mental health) and two global scores: the physical health component summary (PSC-12) and mental health component summary (MSC-12). The scores range from 0 to 100, higher scores equating to a better quality of life.

The neuropathic pain phenotype was assessed by means of the Neuropathic Pain Symptoms Inventory (NPSI). This scale, also adapted and validated for the Spanish population^34^, includes 10 descriptors for quantifying the five most relevant clinical dimensions of neuropathic pain syndrome: evoked pain, spontaneous deep pain, spontaneous superficial pain, paroxysmal pain and paraesthesia/dysthesia. These dimensions are scored on a range from 0 (lack of this pain phenotype) to 10 (maximum presence of this pain phenotype).

### Statistical analysis

A descriptive analysis was performed using absolute (n) and relative (%) frequencies in the case of qualitative variables and measures of centralisation (mean) and dispersion (standard deviation (SD)) in the case of the quantitative variables. An analysis was conducted to compare the sociodemographic and clinical variables between the two groups of patients (with and without DNP), and the total score on the TYM test, the score on all its dimensions and the presence of impairment (TYM score ≤ 41) in both groups.

In the patients with DNP, the variables associated with cognitive function were analysed (both the total TYM score and dichotomized based on a score ≤ 41). According to the type and distribution of the variables, assessed using the Kolmogorov-Smirnoff test, Chi square test, t tests, ANOVA, Mann Whitney U, Kruskal Wallis and correlation coefficients (Pearson or Spearman) were used. In addition, two models were built: a multiple linear regression model, the dependent variable being the total TYM score; and a binary logistic regression model, the dependent variable being the presence/absence of cognitive impairment. The independent variables included in both models were depression, anxiety, sleep, quality of life, and the sociodemographic and clinical variables described above (Table 4). The stepwise method was used for selecting the variables of the models, and R^2^ was considered as the goodness-of-fit measure in the case of the multiple linear regression, and the Hosmer–Lemeshow Chi-square statistic for the logistic regression model. In the multiple linear regression model, tolerance and the variance inflation factor (VIF) were also computed. We assumed that collinearity was not present when the VIF value was below 5 and the tolerance score over 0.2. The analyses were performed with the IBM SPSS v.24 statistical package.

## Results

### Characteristics of the subjects with and without DNP

Among the 149 subjects with neuropathy finally included in the study, 71 had DNP. The patients with DNP were younger (69.55; SD = 9.74 vs. 73.35; SD = 8.20, p = 0.019), had completed primary education more frequently (52.1% vs. 39.7%), and there was a higher percentage that were unemployed or homemakers (40.8% vs. 32%), although in the two latter cases the differences were not significant (Table 1).

**Table 1:** Characteristics of the patients with and without DNP.

Variables	With DNP (N = 71)	Without DNP (N = 78)	p
**Sociodemographic data**	**n (%)**	**n (%)**	
Gender			
Men	36 (50.7%)	47 (60.3%)	0.241^a^
Women	35 (49.3%)	31 (39.7%)	
Age	Mean (SD)	Mean (SD)	0.019^b^
	69.55 (9.74)	73.35 (8.20)	
Education level			
No education	22 (31%)	29 (37.2%)	0.410^c^
Primary studies	37 (52.1%)	31 (39.7%)	
Secondary and University studies	12 (16.9%)	18 (23.1%)	
Employment status			
Unemployed	5 (7%)	3 (3.8%)	0.570^c^
Homemaker	24 (33.8%)	22 (28.2%)	
Working	4 (5.6%)	0 (0%)	
Retired	25 (35.2%)	40 (51.3%)	
Partial and total disability	13 (18.3%)	13 (16.7%)	
**Clinical data**			
Time since type-2 diabetes mellitus diagnosis (years) (N = 140)	Mean (SD)	Mean (SD)	
	11.50 (3.34)	11.68 (3.63)	0.595^b^
HbA1c registered (N = 146)	Mean (SD)	Mean (SD)	
	7.64 (1.60)	7.41 (1.36)	0.367^b^
Medication affecting cognition			
Yes	51 (71.8%)	37 (47.4%)	0.002^a^
Medication for sleep (N = 142)			
Yes	38 (55.9%)	22 (29.7%)	0.002^a^
Medication for pain (N = 142)			
Yes	51 (75.0%)	34 (45.9%)	0.001^a^
Treatment with insulin			
Yes	44 (62.0%)	33 (42.3%)	0.016^a^
Physical comorbidity			
Yes	60 (84.5%)	67 (85.9%)	0.811^a^
History of anxiety			
Yes	22 (30.0%)	10 (12.8%)	0.007^a^
History of depression			
Yes	19 (26.8%)	13 (16.7%)	0.134^a^
**Associated complications**			
Complications			
Yes	50 (70.4%)	52 (66.7%)	0.622^a^
Number complications			
0	21 (44.7%)	26 (55.3%)	0.961^a^
1	25 (46.3%)	29 (53.7%)	
2	18 (51.4%)	17 (48.6%)	
3	5 (54.5%)	5 (45.5%)	
4	1 (50.0%)	1 (50.0%)	
Diabetic retinopathy			
Yes	18 (25.4)	20 (25.6%)	0.968^a^
Diabetic nephropathy			
Yes	15 (21.1%)	16 (20.5%)	0.927^a^
Diabetic foot			
Yes	15 (21.1%)	16 (20.5%)	0.927^a^
Cardiovascular disease			
Yes	33 (46.5%)	29 (37.2%)	0.250^a^
**Cardiovascular risk factors**			
Obesity (> 30 BMI)			
Yes	40 (56.3%)	20 (25.6%)	< 0.001^a^
AHT (N = 148)			
Yes	49 (69.0%)	57 (74.0%)	0.499^a^
Dyslipidaemia (N = 148)			
Yes	48 (68.6%)	40 (51.3%)	0.032^a^
Scales	Mean (SD)	Mean (SD)	
HADS Anxiety score	8.84 (5.46)	3.52 (3.64)	< 0.001^b^
HADS Depression score	8.42 (5.30)	4.15 (3.76)	< 0.001^b^
Index 9	47.03 (23.66)	27.09 (18.14)	< 0.001^b^
US Standardized physical component	32.75 (11.50)	42.79 (11.88)	< 0.001^b^
US Standardized mental component	42.93 (14.42)	53.42 (11.47)	< 0.001^b^
TYM total Score	41.46 (6.70)	41.97 (5.50)	0.994^b^
TYM categorical			
With Cognitive dysfunction	29 (40.8%)	34 (43.6%)	0.735^a^

^a^Pearson’s chi-squared.

^b^Mann–Whitney.

^c^Likelihood ratio.

^d^Student T. SD = Standard Deviation; BMI = Body Mass Index; AHT = Arterial hypertension.

Likewise, the percentage of the patients with obesity (56.3% vs. 25.6%, p =  < 0.001) and dyslipidaemia (68.6% vs. 51.3%, p = 0.032) was higher in the group with DNP. Also of note was that approximately 70% of the subjects in both groups had at least one complication of DM (Table 1).

The patients with DNP obtained higher scores for anxiety (8.84; SD = 5.46 vs. 3.52; SD = 3.64, p =  < 0.001) and depression (8.42; SD = 5.30 vs. 4.15; SD = 3.76, p =  < 0.001), and lower scores on both physical (32.75; SD = 11.50 vs. 42.79; SD = 11.88, p =  < 0.001) and mental components (42.93; SD = 14.42 vs 53.42; SD = 11.47, p =  < 0.001) of the SF-12; they also scored higher on the Index 9 (47.03; SD = 23.66 vs. 27.09; SD = 18.14, p =  < 0.001), indicating worse sleep quality (Table 1).

**Figure 1:**
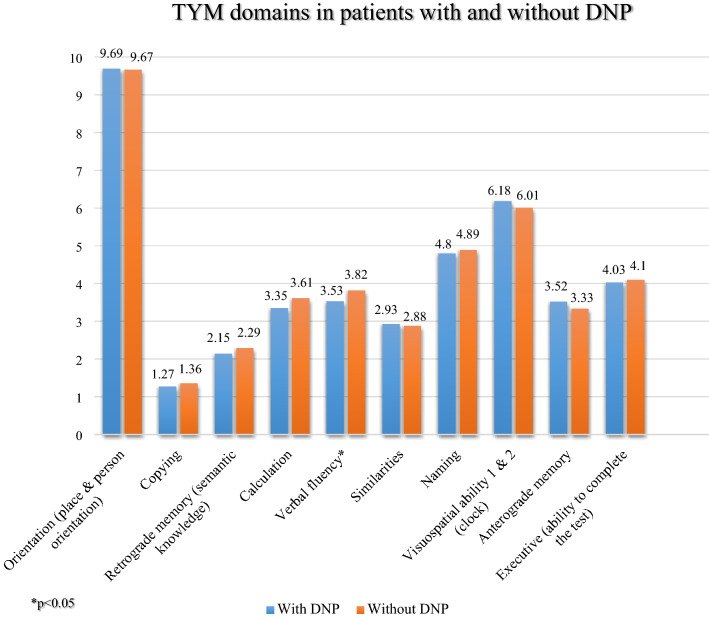
TYM domains in patients with and without DNP.

The results of the TYM showed a lower percentage of patients with cognitive impairment in DNP patients (40.8% vs. 43.6%), although the result was not statistically significant (p = 0.735). Likewise, the mean scores of both groups on this scale were similar (41.46; SD = 6.70 vs. 41.97; SD = 5.50, p = 0.994). Regarding the dimensions of the TYM, the only difference observed was in the verbal fluency, the patients with DNP scoring slightly lower (3.53; SD = 0.98 vs. 3.82; SD = 0.66, p = 0.013) (Fig. 1).

Regarding treatments, it is worth highlighting that the patients with DNP were likely to be taking medication affecting cognition (71.8% vs. 47.4%, p = 0.002), drugs for sleeping (55.9% vs. 29.7%, p = 0.002) and for pain (75.0% vs. 45.9%, p = 0.001) and were under treatment with insulin (62.0% vs. 42.3%, p = 0.016). Moreover, the patients with DNP were also more likely to have a previous history of anxiety (30.0% vs. 12.8%, p = 0.007) than those without (Table 1).

The mean pain intensity score among the patients with DNP was 6.89 and the sensory phenotypes showing the highest mean results were: Paroxysmal Pain = 4.48 (SD = 3.06) and Paraesthesia/Dysesthesia = 5.05 (SD = 2.85).

### Factors associated with cognitive function assessed using the global score of the TYM scale in patients with DNP

The bivariate analysis showed that the women (42.22; SD = 6.67 vs. 40.68; SD = 6.69, p = 0.256), the older patients (r = − 0.367, p = 0.002) and those with a lower educational level obtained lower scores on the TYM (greater cognitive impairment) (Table 2). In addition, a negative correlation was found between the duration of the diabetes and the score on the TYM (r = − 0.277, p = 0.023) (Table 2). The same was found for the depression scores, where a higher score on the HADS-D scale correlated with a lower TYM score (r = − 0.265, p = 0.025). No differences were found in the scores from the scale according to the presence of cardiovascular risk factors, and neither were there correlations with the sensory phenotypes and sleep disorders (Table 2).

**Table 2:** Factors related to the cognitive function (global score of Test Your Memory) in DNP patients. Bivariate analysis.

Variables N = 71	TYM DNP	p
**Sociodemographic data**	**Mean (SD)**	
Gender		
Men	42.22 (6.67)	0.256^a^
Women	40.68 (6.69)	
Age	r = − 0.367	0.002^b^
Education level		
No education	37.95 (7.68)	0.001^c^
Primary studies	41.94 (5.84)	
Secondary studies	46.40 (2.83)	
University studies	46.50 (4.94)	
Employment status		
Unemployed	41.80 (6.72)	0.133^c^
Homemaker	40.45 (5.19)	
Working	43.25 (7.41)	
Retired	40.12 (8.48)	
Partial disability	48.00 (−)	
Total disability	45.00 (3.95)	
**Clinical data**		
Time since type-2 diabetes mellitus diagnosis (years) (N = 100)	r = − 0.277	0.023^b^
HbA1c registered (N = 70)	r = 0.129	0.286^b^
Medication for cognition		
Yes	41.76 (6.27)	0.663^a^
No	40.70 (7.81)	
Medication for sleep (N = 68)		
Yes	41.68 (6.60)	0.985^a^
No	42.27 (5.30)	
Medication for pain (N = 68)		0.439^a^
Yes	41.60 (6.03)	
No	42.94 (5.58)	
Treatment with insulin		
Yes	42.09 (5.91)	0.416^a^
No	40.44 (7.83)	
Physical comorbidity		
Yes	41.57 (6.70)	0.943^a^
No	40.90 (8.31)	
History of anxiety		
Yes	41.59 (6.53)	0.975^a^
No	41.40 (6.84)	
History of depression		
Yes	41.05 (7.08)	0.696^a^
No	41.61 (6.61)	
**Associated complications**		
Complications		
Yes	41.28 (6.89)	0.728^a^
No	41.90 (6.34)	
Number complications		
0	41.90 (6.35)	0.069^c^
1	38.88 (7.73)	
2	44.61 (3.45)	
3	40.50 (8.04)	
4	46.00 (−)	
Diabetic retinopathy		
Yes	42.22 (5.85)	0.569^a^
No	41.20 (6.99)	
Diabetic nephropathy		
Yes	41.26 (5.62)	0.606^a^
No	41.51 (7.00)	
Diabetic foot		
Yes	43.73 (7.59)	0.027^a^
No	40.85 (6.37)	
Cardiovascular disease		
Yes	41.57 (6.86)	0.795^a^
No	41.36 (6.64)	
**Cardiovascular risk factors**		
Obesity (>30 BMI)		
Yes	40.80 (6.91)	0.312^a^
No	42.32 (6.42)	
AHT (N = 69)		0.229^a^
Yes	42.36 (5.52)	
No	39.45 (8.58)	
Dyslipidaemia (N = 70)		
Yes	42.37 (5.49)	0.332^a^
No	39.72 (8.69)	
**Neuropathic Pain Symptom (NPSI) dimensions**		
Pain duration (years)	r = − 0.116	0.334^b^
Pain intensity	r = 0.009	0.938^b^
Evoked Pain	r = 0.010	0.933^b^
Deep Spontaneous Pain	r = − 0.044	0.714^b^
Superficial Spontaneous Pain	r = − 0.009	0.940^b^
Paroxysmal Pain	r = 0.005	0.970^b^
Paraesthesia/Dysesthesia	r = − 0.153	0.203^b^
**Scales**		
HADS Anxiety score	r = 0.067	0.577^b^
HADS Depression score	r = − 0.265	0.025^b^
Index 9	r = 0.046	0.702^b^
US Standardized physical component	r = 0.173	0.150^b^
US Standardized mental component	r = 0.105	0.383^b^

Bivariate analysis.

^a^Mann–Whitney.

^b^Spearman Correlation Coefficient.

^c^Kruskal-Wallis. SD = Standard Deviation; BMI = Body Mass Index; AHT = Arterial hypertension.

The multivariate analysis showed that the older patients (B = − 0.258, p = 0.001), and those under treatment with insulin (B = − 2.919, p = 0.060) presented lower TYM scores (worse cognitive function). By contrast, the patients with AHT (B = 3.291, p = 0.025), those with primary studies (B = 4.359, p = 0.010) and secondary or university studies (B = 8.369, p = 0.001), and those with higher scores on the physical component of the SF-12v2 (B = 0.118, p = 0.044) scored higher on the TYM (better cognitive function). Taking medication that affected cognition was included as an adjustment variable in the model. Analysing the tolerance and VIF values, collinearity was not present between the variables included in the model (Table 4).

### Factors associated with the presence of cognitive impairment (TYM score ≤ 41) in patients with DNP

The bivariate analysis showed that impairment was more common in the women vs men (45.70% vs 36.10%, p = 0.441), in the older subjects (73.343 vs. 66.93, p = 0.013), and in those without studies (72.70%) (Table 3).

**Table 3:** Factors related to cognitive impairment (Test Your Memory ≤ 41) in DNP patients. Bivariate analysis.

Variables N = 71	With Cognitive impairment	Without Cognitive impairment	p
**Sociodemographic data**	**n (%)**	**n (%)**	
Gender			
Men	13 (36.1%)	23 (63.9%)	0.441^a^
Women	16 (45.7%)	19 (54.3%)	
Age	Mean (SD)	Mean (SD)	0.013^b^
	73.34 (7.51)	66.93 (10.31)	
Education level			
No education	16 (72.7%)	6 (27.3%)	0.000^c^
Primary studies	13 (35.1%)	24 (64.9%)	
Secondary studies	0 (0.0%)	10 (100.0%)	
University studies	0 (0.0%)	2 (100.0%)	
Employment status			
Unemployed	2 (40.0%)	3 (60.0%)	0.296^c^
Homemaker	12 (50.0%)	12 (50.0%)	
Working	1 (25.0%)	3 (75.0%)	
Retired	12 (48,0%)	13 (52.0%)	
Partial disability	0 (0.0%)	1 (100.0%)	
Total disability	2 (16.7%)	10 (83.3%)	
**Clinical data**			
Time since type-2 diabetes mellitus diagnosis (years) (N = 67)	Mean (SD)	Mean (SD)	
	12.44 (3.30)	10.78 (3.22)	0.015^b^
HbA1c registered (N = 70)	Mean (SD)	Mean (SD)	
	7.75 (1.67)	7.57 (1.54)	0.849^b^
Medication for cognition			
Yes	19 (65.5%)	32 (76.2%)	0.326^a^
Medication for sleep (N = 68)			
Yes	13 (48.1%)	25 (61.0%)	0.297^a^
Medication for pain (N = 68)			
Yes	21 (77.8%)	30 (73.2%)	0.668^a^
Treatment with insulin			
Yes	16 (55.2%)	28 (66.7%)	0.327^a^
Physical comorbidity			
Yes	25 (86.2%)	35 (83.3%)	0.742^a^
History of anxiety			
Yes	8 (27.6%)	14 (33.3%)	0.607^a^
History of depression			
Yes	9 (31%)	10 (23.8%)	0.499^a^
**Associated complications**			
Complications			
Yes	21 (42.0%)	29 (58.0%)	0.760^a^
Number complications			
0	8 (38.1%)	13 (61.9%)	0.169^c^
1	14 (56.0%)	11 (44.0%)	
2	4 (22.2%)	14 (77.8%)	
3	3 (50.0%)	3 (50.0%)	
4	0 (0.0%)	1 (100.0%)	
Diabetic retinopathy			
Yes	7 (38.9%)	11 (61.1%)	0.845^a^
Diabetic nephropathy			
Yes	7 (46.7%)	8 (53.3%)	0.606^a^
Diabetic foot			
Yes	4 (26.7%)	11 (73.3%)	0.208^a^
Cardiovascular disease			
Yes	13 (39.4%)	20 (60.6%)	0.817^a^
**Cardiovascular risk factors**			
Obesity (>30 BMI)			
Yes	19 (47.5%)	21 (52.5%)	0.195^a^
AHT			
Yes	18 (36.7%)	31 (63.3%)	0.293^a^
Dyslipidaemia (N = 70)			
Yes	17 (35.4%)	31 (64.6%)	0.248^a^
Neuropathic Pain Symptom (NPSI) dimensions	Mean (SD)	Mean (SD)	
Pain duration (years)	5.14 (3.37)	3.84 (3.30)	0.071^b^
Pain intensity	7.00 (2.42)	6.81 (2.45)	0.855^b^
Evoked Pain	3.00 (2.78)	2.70 (2.37)	0.700^b^
Deep Spontaneous Pain	4.00 (3.36)	3.36 (3.03)	0.474^b^
Superficial Spontaneous Pain	4.31 (4.43)	2.90 (3.42)	0.163^b^
Paroxysmal Pain	4.56 (3.30)	4.41 (2.92)	0.760^b^
Paraesthesia/Dysesthesia	5.67 (2.73)	4.61 (2.87)	0.163^b^
Scales	Mean (SD)	Mean (SD)	
HADS Anxiety score	9.37 (5.58)	8.47 (5.42)	0.534^b^
HADS Depression score	9.93 (5.47)	7.38 (4.99)	0.043^b^
Index 9	45.13 (21.67)	48.34 (25.12)	0.527^b^
US Standardized physical component	30.72 (12.03)	34.15 (11.04)	0.131^b^
US Standardized mental component	41.11 (15.12)	44.19 (13.96)	0.343^b^

^a^Pearson’s chi-squared.

^b^Mann–Whitney.

^c^Likelihood ratio; SD = Standard Deviation; BMI = Body Mass Index; AHT = Arterial hypertension.

Likewise, in the patients with cognitive impairment (TYM score ≤ 41), the time since the diagnosis of diabetes was greater (14.44; SD = 3.30 vs. 10.78; SD = 3.22, p = 0.015), and they obtained higher scores on the HADS depression (9.93; SD = 5.47 vs. 7.38; SD = 4.99, p = 0.043) and anxiety scales (9.37; SD = 5.58 vs. 8.47; SD = 5.42, p = 0.534) (Table 3).

Regarding the sensory phenotypes of pain, the subjects with cognitive impairment has a higher mean score for superficial spontaneous pain (4.31; SD = 4.43 vs. 2.90; SD = 3.42, p = 0.163) and paraesthesia/dysesthesia (5.67; SD = 2.73 vs. 4.61; SD = 2.87, p = 0.163) (Table 3).

The multivariate analysis (TYM score ≤ 41) (Table 4) showed that the obese patients (OR = 17.277; CI95%: 2.000–149.270, p = 0.011) and those with a longer diabetes duration (OR = 1.317; CI95%: 1.001–1.733, p = 0.049) had a greater risk of cognitive impairment. On the other hand, a higher score on the MOS-9 index (worse quality of sleep) (OR = 0.944; CI95%: 0.898–0.992, p = 0.024), a higher score on the SF-12 (OR = 0.911; CI95%: 0.837–0.992, p = 0.043), having primary studies vs not having studies (OR = 0.055; CI95%: 0.007–0.431, p = 0.006) and taking medication for sleep (OR = 0.069; CI95%: 0.009–0.541, p = 0.011) were factors associated with a lower risk of cognitive impairment. The age variable, although not significant, remained in the model as it was considered to be an adjustment variable (Table 4). Taking drugs with an effect on cognitive function was initially included in the model, but was eventually eliminated due to the presence of the covariable *drugs for sleep*, with which it shares a lot of information.

**Table 4:** Factors related to cognitive function (Test Your Memory) in DNP patients. Multivariate analysis.

Model 1 TYM score: multiple linear regression model						
Variable	Category/Unit	B (SE)	95%CI	p-value	Tolerance	VIF
Constant		49.909 (5.853)	(37.814 to 61.356)	0.000		
AHT	Yes No*	3.291 (1.465)	(0.446 to 6.351)	0.025	0.929	1.076
Education level (Ref. No education)	Primary studies	4.359 (1.664)	(1.113 to 78.09)	0.010	0.617	1.620
	Secondary and University studies	8.369 (2.288)	(4.119 to 14.150)	0.001	0.580	1.724
Age		− 0.258 (0.071)	(− 0.400 to − 0.115)	0.001	0.902	1.109
PCS	US standardized physical component scale	0.118 (0.059)	(0.003 to 0.241)	0.044	0.938	1.066
Insulin therapy	Yes No*	− 2.919 (1.629)	(− 6.571 to 0.145)	0.060	0.682	1.466
Medication for cognition	Yes No*	2.061 (1.540)	(− 0.834 to 5.523)	0.145	0.889	1.125
Dependent variable: TYM- Total Score B, Beta; SE, Standard error; CI, Confidence interval; R^2^ = 0.325						

Model 2 Presence of cognitive impairment TYM score ≤ 41: Binary Logistic regression model						
Variables	Category/Unit	B (SE)	Wald-Statistic	OR	95%CF	p-value
Constant		2.438 (4.570)	0.285	11.455		0.594
MOS score, I-9		− 0.058 (0.026)	5.089	0.944	(0.898 to 0.992)	0.024
MCS	US standardized Mental component scale	− 0.093 (0.043)	4.592	0.911	(0.837 to 0.992)	0.043
Education level (reference category: No education	Primary studies	− 2.895 (1.048)	7.633	0.055	(0.007 to 0.431)	0.006
	Secondary and University studies	− 26.393 (9261.390)	0.000	0.000	0.000–	0.998
Obesity	Yes No*	2.849 (1.100)	6.707	17.277	(2.000 to 149.270)	0.011
Medication for sleep	Yes No*	− 2.676 (1.052)	6.471	0.069	(0.009 to 0.541)	0.011
Age		0.041 (0.044)	0.862	1.042	(0.955 to 1.137)	0.353
Time since type-2 diabetes mellitus diagnosis (years)		0.275 (0.140)	3.860	1.317	(1.001 to 1.733)	0.049
N = 64; Dependent variable: Presence/Absence* of Cognitive impairment. Homer and Lemeshow: *X*^2^ = 4.158; p-value = 0.843. B, Beta; SE, Standard error; OR, Odd Ratio; CI, Confidence interval.						
Variables included in the initial models: Gender, Age, Education level, Employment status, Time since type-2 diabetes mellitus diagnosis, HbA1c, Medication for cognition, Medication for sleep, Medication for pain, Insulin therapy, History of anxiety, History of depression, Physical comorbidity, Complications, Diabetic retinopathy, Diabetic nephropathy, Diabetic foot, Cardiovascular disease, Obesity, AHT, Dyslipidaemia, Pain duration, Pain intensity, HADS Anxiety, HADS Depression, MOS score, I-9, PCS and MCS.						

## Discussion

This study analyses the differences in cognitive function and its dimensions in patients with T2DM with and without DNP and identifies factors associated with cognitive function in patients with DNP. Of note among the results obtained is that, although the mean scores on the TYM scales were slightly lower than the cut-off point established by Brown et al.^26^, they were similar in both groups. Likewise, no significant differences were observed in the frequency of cognitive impairment between the two groups of patients analysed.

Despite the lack of differences in cognitive function observed between groups, the study found that the subjects with DNP were more frequently under treatment with insulin, suffered from worse obesity and dyslipidaemia, had higher levels of anxiety and depression, worse quality of sleep and more frequently took medication for sleep, pain and drugs that affected cognition than the patients without DNP. At this vein, it is well known that DNP is a chronic disease associated with long-term suffering and disability, and with significant interference in daily life^17^. It can explain the observed significant impact on mental health, quality of life and sleep in DNP patients, compared to those without DNP, as shown in several studies^16,17,35^. Regarding the results observed for cognitive function, other authors^13^ report that patients with T2DM, both with and without DNP, may present cognitive impairment. In this sense, Zhang et al.^36^ suggest that oxidative stress and neuronal degeneration brought about by greater glycaemic variability are associated with a greater presence of diabetic neuropathy and cognitive impairment.

A noteworthy result is the fact that the subjects with DNP performed worse in verbal fluency that those without DNP. Verbal fluency depends on the correct functioning of the prefrontal cortex, a region that could be affected by pain processing, which could explain the results observed in our study^37^. However, these data should be interpreted with caution, as they relied exclusively on the TYM measure. Additional tests evaluating these domains are recommended for future studies.

Analysing the factors associated with cognitive function in the patients with DNP, those that were older and those under treatment with insulin obtained lower scores on the TYM scale (more impairment). In addition, the presence of cognitive impairment (TYM score ≤ 41) was associated with the presence of obesity and a longer duration of diabetes and with the presence of depression.

Regarding age, several studies^1,2^ have shown an inverse relationship with cognitive functioning. Similarly, DNP and diabetes have been reported to be considerably more prevalent among older people^9^, evidence existing that patients suffering from CP and diabetes presenting worse cognitive functioning. In the case of diabetes, this impairment is related with an increase in insulin resistance, greater fluctuations of glucose and high levels of HbA1c^38^; as for chronic pain, it is associated with the interference produced by the pain in important regions for cognitive performance such as the prefrontal cortex and the hippocampus^7^.

With respect to insulin treatment, some authors highlight that the patients receiving this treatment most frequently have the disease the longest and present worse metabolic control, which, as mentioned above, are factors associated with impaired cognitive function in individuals with diabetes^38^. The duration of the disease and obesity were also factors related with the presence of impaired cognitive function in the patients with DNP, although these results were not evident when the TYM score was considered as the dependent variable; this is possibly due to the increase produced on the scale not being enough to reach statistical significance and to it being variable, as deduced by the wide 95%CI of the results.

The relation between cognitive impairment and depression found in the study has been shown in previous studies carried out in diabetic patients with peripheral neuropathy. In this line, a recently published meta-analysis^39^ shows that this relationship could be partly due to psychosocial stress produced for suffering a chronic illness^4^. Likewise, some studies have shown that depression, which is present in 30–60% of pain patients, can increase the risk of cognitive impairment due the neuroinflammation and brain atrophy that this disorder can produce^7^. Furthermore, the presence of pain in these patients is related to loss of functional capacity and social contacts that also increase the risk of depressive symptoms^40^. All of these factors can lead to a decrease of self-sufficiency^5,40^, and consequently to decrease cognitive function asreported by Calatayud et al.^41^.

The relationship observed between cognitive function and level of education is in agreement with that found in other studies^40^, where a higher level of education was related with a greater cognitive reserve^42^. Moreover, it has been shown that the higher the educational level, the better the control and knowledge of the disease, and self-care practices and adherence to treatment is better, which are factors related with maintaining cognitive functioning in persons with diabetes^43,44^. Another result of our study, again in line with the literature, is that higher scores on the mental and physical component of quality of life are associated with better cognitive function in patients with DNP. Better physical and mental health means less limitations in performing daily activities, greater autonomy and a greater chance of the patients conducting rewarding activities, which has been shown to stimulate cognitive functioning^45,46^.

Unexpected results were the inverse relationship between cognitive function and AHT (multiple linear regression model) or that found with quality of sleep (binary logistic regression model).

Generally, AHT is a risk factor that is often related with the presence of diabetes and cognitive impairment^5^. However, other studies have reported the opposite to be true^47^. More specifically, Ruitenberg et al.^48^ in a longitudinal study of subjects that ultimately developed dementia found an inverse relationship between AHT and dementia. Along these lines, den Heijer et al.^49^ also observed that the cortical atrophy produced in Alzheimer’s disease is related with a decrease in blood pressure levels. Moreover, on the basis of taking medication for hypertension being associated with less cognitive impairment^47^, a possible hypothesis for explaining our results is that patients diagnosed with AHT take medication for it and are therefore more controlled, unlike those without a diagnosis and who logically are not under treatment for it. However, no information was included in this study about taking medication for hypertension, making it impossible to prove this hypothesis.

Finally, although sleep disorders are common among persons with diabetes with DNP^16^, and other studies have shown them to be related with impaired cognitive performance^40^, this relationship was not observed in this study. One explanation could be the different instruments used in these studies, the Pittsburgh Sleep Quality Index often being used^50^. Moreover, our results are based on the Index 9, a complex index constructed using the sum of different items that comprise the dimensions in the MOS scale, where we observe inverse relationships with the TYM in the scores of these dimensions. For example, better results are obtained among subjects with impairment in the sleep disturbance and adequacy of sleep dimensions; however, among the subjects without cognitive impairment, the results on the shortness of breath dimension are better (data not shown).

Taking hypnotic medication was associated with a lower risk of impairment, as expected, as sleep-enhancing drugs have been shown to mitigate cognitive impairment if the kind used and the doses prescribed are controlled^51^. The frequency of taking these drugs was high among the patients with DNP, which could influence the result observed on the Index-9, although information about the type and dose was not collected in the study.

### Strengths and limitations

As strengths, we highlight the use of validated scales that enable better information to be obtained from the population analysed. Likewise, the multi-centred design allowed us to obtain a more representative sample, although it was not possible to reach the sample size initially calculated due to the Covid-19 pandemic, which forced us to put an end to the data collection earlier than planned. Although this is a limitation that could diminish the power of the study, the information provided by the study is still relevant. Another strength is that we have analysed the cognitive domains of the TYM separately in order to obtain more detailed information about the dimensions that encompass cognitive function, an innovative topic that has not been studied before, to our knowledge.

As a limitation, it is necessary to highlight that the cross-sectional design of the study does not allow causal relationships to be established between cognitive function and the variables studied. Furthermore, although some authors recommend multiple instruments to more reliably assess the dimensions of cognitive function, we only use TYM to ensure that the assessment session did not take too long. The TYM scale has been translated, adapted and validated in Spanish in patients with chronic pain included neuropathic pain by our research group^27,28^. Another limitation is our specific selection of high-risk patients, which could bias the sample in favor of those with more severe mood and sleep disorders, and worse cognitive function. However, we decided to choose this group of patients because they were more likely to suffer from diabetic neuropathy and DNP, making the study more efficient.

## Conclusions

This study shows that patients with T2DM, both with and without DNP, present cognitive impairment. No greater risk was observed when the pain was more intense or according to the sensory profile of the patients. In addition, it is noteworthy that being older, treatment with insulin, obesity, a longer duration of diabetes and the presence of depression were associated with a greater risk of cognitive impairment in patients with DNP. While a higher level of education, a better mental and physical component of quality of life, a higher AHT and a poor quality of sleep were associated with a better cognitive function in patients with DNP. Identifying and controlling these factors should be an essential intervention for maintaining the cognitive function of patients with T2DM and DNP.

## Data Availability

The datasets analysed during the current study are available from the corresponding author on reasonable request.

## References

[1] Jongsiriyanyong S, Limpawattana P (2018). Mild cognitive impairment in clinical practice: A review article. Am. J. Alzheimer’s Dis. Demen..

[2] Vega-Alonso T (2018). Prevalence of cognitive impairment in Spain: The Gómez de Caso study in health sentinel networks. Neurologia.

[3] Jongsiriyanyong S, Limpawattana P (2018). Mild cognitive impairment in clinical practice: A review article. Am. J. Alzheimer’s Dis. Other Demen..

[4] Van Sloten TT, Sedaghat S, Carnethon MR, Launer LJ, Stehouwer CDA (2020). Cerebral microvascular complications of type 2 diabetes: Stroke, cognitive dysfunction, and depression. Lancet Diabetes Endocrinol..

[5] Li W, Sun L, Li G, Xiao S (2019). Prevalence, influence factors and cognitive characteristics of mild cognitive impairment in type 2 diabetes mellitus. Front. Aging Neurosci..

[6] Whitlock EL (2017). Association between persistent pain and memory decline and dementia in a longitudinal cohort of elders. JAMA Intern. Med..

[7] Bell T, Franz CE, Kremen WS (2021). Persistence of pain and cognitive impairment in older adults. J. Am. Geriatr. Soc..

[8] Achterberga W, Lautenbacherb S, Huseboc B, Erdalc A, Herr K (2020). Pain in dementia. Pain.

[9] Shillo P (2019). Painful and painless diabetic neuropathies: What is the difference?. Curr. Diab. Rep..

[10] Rosenberger DC, Blechschmidt V, Timmerman H, Wolff A, Treede RD (2020). Challenges of neuropathic pain: Focus on diabetic neuropathy. J. Neural Transm..

[11] Pop-Busui R (2017). Diabetic neuropathy: A position statement by the American diabetes association. Diabetes Care.

[12] Sloan G (2018). A new look at painful diabetic neuropathy. Diabetes Res. Clin. Pract..

[13] Elsharkawy RE (2021). Peripheral polyneuropathy and cognitive impairment in Type II diabetes mellitus. Neuropsychiatr. Dis. Treat..

[14] Naranjo C, Ortega-Jiménez P, del Reguero L, Moratalla G, Failde I (2020). Relationship between diabetic neuropathic pain and comorbidity. Their impact on pain intensity, diabetes complications and quality of life in patients with type-2 diabetes mellitus. Diabetes Res. Clin. Pract..

[15] Selvarajah D (2014). Magnetic resonance neuroimaging study of brain structural differences in diabetic peripheral neuropathy. Diabetes Care.

[16] Naranjo C (2019). Anxiety, depression and sleep disorders in patients with diabetic neuropathic pain: A systematic review. Expert Rev. Neurother..

[17] Kioskli K, Scott W, Winkley K, Kylakos S, McCracken LM (2019). Psychosocial factors in painful diabetic neuropathy: A systematic review of treatment trials and survey studies. Pain Med. (US).

[18] Bobo WV (2022). Association of depression and anxiety with the accumulation of chronic conditions. JAMA Netw. Open.

[19] Haack M, Simpson N, Sethna N, Kaur S, Mullington J (2020). Sleep deficiency and chronic pain: Potential underlying mechanisms and clinical implications. Neuropsychopharmacology.

[20] Orgeta V (2022). Psychological treatments for depression and anxiety in dementia and mild cognitive impairment. Cochrane Datab. Syst. Rev..

[21] American Diabetes Association. 2. Classification and diagnosis of diabetes: Standards of medical care in diabetes-2021. *Diabetes Care* **44**, S15–S33 (2021). 10.2337/dc21-S002 33298413

[22] Association AD (2016). 2. Classification and diagnosis of diabetes. Diabetes Care.

[23] Zhang Q (2018). Easier operation and similar power of 10 g monofilament test for screening diabetic peripheral neuropathy. J. Int. Med. Res..

[24] Perez C (2007). Validity and reliability of the Spanish version of the DN4 (Douleur Neuropathique 4 questions) questionnaire for differential diagnosis of pain syndromes associated to a neuropathic or somatic component. Health Qual. Life Outcomes.

[25] Brown J, Pengas G, Dawson K, Brown LA, Clatworthy P (2009). Self administered cognitive screening test (TYM) for detection of Alzheimer’s disease: Cross sectional study. BMJ.

[26] Brown J (2019). Test Your Memory (TYM test): Diagnostic evaluation of patients with non-Alzheimer dementias. J. Neurol..

[27] Ojeda B (2016). Assessing the construct validity and internal reliability of the screening tool test your memory in patients with chronic pain. PLoS ONE.

[28] Ojeda B, Salazar A, Dueñas M, Failde I (2012). Traducción y adaptación al castellano del Cuestionario de Detección de Trastorno Cognitivo Leve. Med. Clin..

[29] Selvarajah D (2014). The contributors of emotional distress in painful diabetic neuropathy. Diab. Vasc. Dis. Res..

[30] Herrero MJ (2003). A validation study of the hospital anxiety and depression scale (HADS) in a Spanish population. Gen. Hosp. Psychiatry.

[31] Quintana JM (2003). Evaluation of the psychometric characteristics of the Spanish version of the Hospital Anxiety and Depression Scale. Acta Psychiatr. Scand..

[32] Viala-Danten M, Martin S, Guillemin I, Hays RD (2008). Evaluation of the reliability and validity of the Medical Outcomes Study sleep scale in patients with painful diabetic peripheral neuropathy during an international clinical trial. Health Qual. Life Outcomes.

[33] Schmidt S (2012). Normas de referencia para el Cuestionario de Salud SF-12 versión 2 basadas en población general de Cataluña. Med. Clin..

[34] Villoria J, Rodríguez M, Berro MJ, Stern A, Sánchez-Magro I (2011). Psychometric validation of the neuropathic pain symptom inventory for its use in Spanish. J. Pain Symptom. Manag.

[35] Gylfadottir SS (2020). Diabetic polyneuropathy and pain, prevalence, and patient characteristics: A cross-sectional questionnaire study of 5,514 patients with recently diagnosed type 2 diabetes. Pain.

[36] Zhang X, Yang X, Sun B, Zhu C (2021). Perspectives of glycemic variability in diabetic neuropathy: A comprehensive review. Commun. Biol..

[37] Chou PH (2018). Reduced frontal activity during a verbal fluency test in fibromyalgia: A near-infrared spectroscopy study. J. Clin. Neurosci..

[38] Sharma G (2020). Cognitive impairments in type 2 diabetes, risk factors and preventive strategies. J. Basic Clin. Physiol. Pharmacol..

[39] Palomo-Osuna J, de Sola H, Dueñas M, Moral-Munoz JA, Failde I (2022). Cognitive function in diabetic persons with peripheral neuropathy: A systematic review and meta-analysis. Expert Rev. Neurotherapeut..

[40] Ojeda B, Dueñas M, Salazar A, Mico J, Torres L (2017). Factors influencing cognitive impairment in neuropathic and musculoskeletal pain and fibromyalgia. Pain Med..

[41] Calatayud E, Salavera C, Gómez-Soria I (2021). Cognitive differences in the older adults living in the general community: Gender and mental occupational state study. Int. J. Environ. Res. Public Health.

[42] Moran C, Beare R, Wang W, Callisaya M, Srikanth V (2019). Type 2 diabetes mellitus, brain atrophy, and cognitive decline. Neurology.

[43] Almigbal TH (2019). Association of health literacy and self-management practices and psychological factor among patients with type 2 diabetes mellitus in Saudi Arabia. Saudi Med. J..

[44] RobatSarpooshi D (2020). The relationship between health literacy level and self-care behaviors in patients with diabetes. Patient Relat. Outcome Meas..

[45] Mandolesi L (2018). Effects of physical exercise on cognitive functioning and wellbeing: Biological and psychological benefits. Front. Psychol..

[46] Bai A, Tao L, Huang J, Tao J, Liu J (2021). Effects of physical activity on cognitive function among patients with diabetes in China: A nationally longitudinal study. BMC Public Health.

[47] Iadecola C, Gottesman RF (2019). Neurovascular and cognitive dysfunction in hypertension: Epidemiology, pathobiology, and treatment. Circ. Res..

[48] Ruitenberg A (2001). Blood pressure and risk of dementia: Results from the Rotterdam study and the Gothenburg H-70 study. Dement. Geriatr. Cogn. Disord..

[49] den Heijer T (2003). Association between blood pressure levels over time and brain atrophy in the elderly. Neurobiol. Aging.

[50] Buysse-Charles F, Reynolds-Ill DJ, Monk TH, Berman SR, Kupfer DJ (1988). The Pittsburgh sleep quality index: A new instrument for psychiatric practice and research. Psychiatry Res..

[51] Burke SL (2018). Mild cognitive impairment: Associations with sleep disturbance, apolipoprotein e4, and sleep medications. Sleep Med..

